# Improved Chloride Ion Sensing Performance of Flexible Ag-NPs/AgCl Electrode Sensor Using Cu-BTC as an Effective Adsorption Layer

**DOI:** 10.3389/fchem.2019.00637

**Published:** 2019-09-24

**Authors:** Byungkwan Kwak, Soobin Park, Han-Seung Lee, Jiwon Kim, Bongyoung Yoo

**Affiliations:** ^1^Department of Material Science and Chemical Engineering, Hanyang University, Ansan-si, South Korea; ^2^Department of Architectural Engineering, Hanyang University, Ansan-si, South Korea; ^3^Materials Science and Chemical Engineering Center, Institute for Advanced Engineering, Yongin-si, South Korea

**Keywords:** ion selective sensor, chloride ion sensor, Cu-BTC, Ag nanoparticle, electroless deposition

## Abstract

We designed the flexible chloride ion selective sensor that directly monitors electrochemical reactions of chloride ions without using a reference electrode. A flexible polytetrafluoroethylene (PTFE) substrate was utilized to provide bendability to the fabricated sensor. As an ion selective material, Ag nanoparticles were employed on the MWCNTs loaded on the PTFE substrate. Enhanced adsorption property of the fabricated sensor toward the chloride ions was given by incorporation of hydrophilic copper benzene-1,3,5-tricarboxylate (Cu-BTC) with great flexibility and stability. Accordingly, compared to the bare sensor the sensing performance of the Cu-BTC treated Ag NPs/AgCl electrode sensor was improved by indicating the decrease in response and recovery time about 4 times. It elucidated that the Cu-BTC layer could work as an effective medium between the Ag-NPs surface and electrolyte containing chloride ions. As a result of contact angle measurement, the hydrophilicity much increased in the Cu-BTC treated sensor because the exposed surface of the sensor not treated by the Cu-BTC largely consisted of hydrophobic MWCNTs. Furthermore, the Cu-BTC layer could hold the electrolyte for effective adsorption of analytes with large specific surface area.

## Introduction

Chloride ion sensing is significant in researches such as medical examination (Warwick et al., 1986; Huber et al., 1998; Jiang et al., 1998), environmental monitoring (Elsener et al., 2003; Angst et al., 2010; Harris et al., 2016), and other numerous industrial fields (Badr et al., 1999; Da Silva et al., 2005; Babu et al., 2008). The chloride ion sensing techniques have been utilized in various areas. For example, the chloride ion is indicative of patient's physiological issues in human body. Also, the chloride ion impinges on building durability as a critical degradation factor in drastic corrosion of reinforcing bars in building structures. Therefore, it is important to develop precise and practical chloride ion sensor for its diverse use.

The chloride ion sensor is categorized by a type of signal determination such as electrochemical measurements (Montemor et al., 2006), ion chromatography (Pereira et al., 2008), spectroscopy (Fox and Barker, 1978) where the detections are investigated by monitoring a change of electrochemical potential/current, difference of ionic strength, and absorption spectrum, respectively. Especially, an ion selective electrode sensor, which is one of the electrochemical measurements, is promising because it is intuitive to monitor the presence of chloride ion as a function of its concentration. This is a distinctive merit compared to the ordinary electrochemical sensors that indirectly monitor the presence of chloride ion. For example, reading of corrosion rate of structures by corrosion detecting methods (Elsener et al., 2003; Angst et al., 2010; Harris et al., 2016) is a qualitative analysis and unavoidable to hinder degradation of sensor itself.

The sensing performance of ion selective electrode sensors has been optimized by varying the sensing materials used as the electrode, for instance, silver/silver chloride (Ag/AgCl) ion selective electrode (Montemor et al., 2006), polymeric membrane electrode (Sjöberg-Eerola et al., 2007; Gupta et al., 2009), copper complexes-based sensor (Mahajan et al., 2006), and so on (Zhang et al., 2002; Xu et al., 2004). Among them, the Ag/AgCl electrode sensor is widely investigated owing to excellent selectivity of Ag/AgCl electrode toward chloride ion and high stability in aqueous solutions (Climent-Llorca et al., 1996). The efforts to detect the critical chloride ion content using the Ag/AgCl ion selective electrode have been conducted by understanding a role of diffusion potential in overall sensing environment that led to modification of sensor platforms. In fact, the signal of Ag/AgCl ion selective electrode sensor is mainly determined by reading of liquid junction potential measured by the reference electrode in the system. During this process, unfortunately, there is liquid junction potential errors possibly arisen at the interfaces to hinder the accurate sensing signal. According to Angst and Vennesland the deviation of potentials could be correctable by means of either chemical composition control at the contacting solution similar to the pore solution or accurate positioning of reference electrode (Angst and Vennesland, 2009). Furthermore, the Ag/AgCl electrode sensor is rugged in constructions, therefore, it is physically weak in operating environments. For example, a reference electrode that is inevitable for operation easily shows malfunctions caused by sudden mechanical stress and/or pH changes inside of structures so that those restrict its permanent usage. Therefore, it is important to develop the sensor that is possible for intuitive measurement of chloride ions and non-destructive under extreme environmental conditions.

So as to develop the chloride ion sensor without using the reference electrode, we designed the sensor to directly monitor electrochemical reactions of chloride ions on the Ag/AgCl electrodes. Firstly, a flexible polytetrafluoroethylene (PTFE) substrate was utilized as a way to provide bendability on the fabricated sensor. We employed Ag nanoparticles (NPs) on which the reduction of chloride ion appears by an electroless deposition method on chemically-stable and resistive multi-walled carbon nanotubes (MWCNTs). Instead of the reference electrode, the change of electrical resistance by chloride ion adsorption on the Ag/AgCl electrode read as sensing signal transduced via MWCNTs as a channel.

In addition, the enhanced adsorption property toward the chloride ions was given by incorporation of metal-organic frameworks (MOFs) on the top of the Ag NPs as effective chloride ion adsorption layer. The MOFs, which is self-assembly of organic ligands and charged metal oxide clusters, have been known for the ion adsorption property (Karra and Walton, 2008). Moreover, the great porosity and high internal surface areas take center stage of appropriacy as sensing adsorbents (Pohle et al., 2011; Davydovskaya et al., 2013, 2014a,b; Pentyala et al., 2015). In this work, copper benzene-1,3,5-tricarboxylate (Cu-BTC), which is hydrophilic MOFs with great flexibility and stability (Hamon et al., 2011), was utilized to enhance the ion selective sensor performance by offering proper adsorption capacity and selectivity toward the chloride ions (Chui et al., 1999; Karra and Walton, 2008; Canivet et al., 2014).

Accordingly, compared to the bare sensor the sensing performance of the Cu-BTC treated Ag NPs/AgCl ion selective electrode sensor was improved by indicating the decrease in response and recovery time about 4 times at the same conditions. It elucidated that the Cu-BTC layer would work as an effective medium between the Ag-NPs surface and electrolyte containing chloride ions. As a result of contact angle measurement, the hydrophilicity much increased in the Cu-BTC treated sensor because the exposed surface of the sensor not treated by the Cu-BTC largely consisted of hydrophobic MWCNTs. Furthermore, the Cu-BTC layer would hold the electrolyte for effective adsorption of analytes with large specific surface area.

## Experimental

### Fabrication of Flexible Ag-NPs/AgCl Electrode Sensor

**Figure 1:**
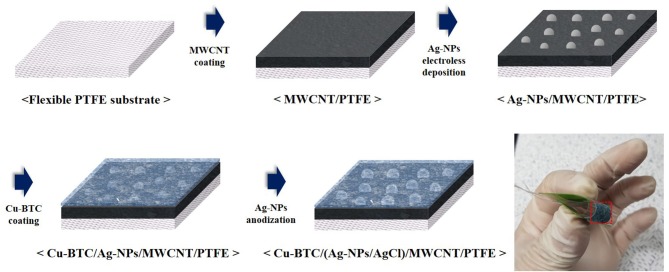
Schematic diagram of fabrication process for a flexible Ag-NPs/AgCl electrode sensor.

Schematic diagram of fabrication process for a flexible Ag-NPs/AgCl electrode sensor was indicated in Figure 1. For the fabrication of chloride ion sensor, the multi-walled carbon nanotubes (MWCNTs) (>90% carbon basis, Sigma-aldrich) were used as conducting channels because of their sensitivity in charge transfer (Musameh and Wang, 2003). As a chloride ion detector material, the Ag nanoparticles were decorated on the MWCNTs with a control of the diameter and density by using the electroless deposition process. For the facile electroless deposition, which is processed in aqueous electrolytes under ambient conditions, the MWCNTs are required to have well-dispersed property in a solution. Therefore, the MWCNTs were functionalized to have carboxyl groups and hydroxyl groups where these functional groups help to prevent the agglomeration of CNTs in the aqueous solution (Osorio et al., 2008). Also, these functional groups facilitate the immobilization of Ag nanoparticles on the CNT surface (Wildgoose et al., 2006; Cheng et al., 2013). The diameter and length of MWCNTs used in this experiment was ranged from 110 to 170 nm and from 5 to 9 μm, respectively. For the functionalization of MWCNTs, 0.1 g of carbon nanotubes were dispersed in the mixture of 15 mL HNO_3_ (60%, Daejung chemicals) and 45 mL H_2_SO_4_ (95%, Daejung chemicals) followed by sonicated at 60°C for 9 h (Osorio et al., 2008). After that, the solution was stored for 24 h at room temperature.

The MWCNTs were deposited on the flexible PTFE substrate via vacuum filtration. Firstly, 3 mL of the functionalized CNT solution was homogeneously dispersed in 200 mL ethanol. Electrical aspirator (VE-11, Lab companion) was used for filtering the solution on a PTFE membrane (Pore size: 0.45 μm, hydrophobic, Chmlab). Finally, the MWCNTs deposited PTFE was neutralized by distilled water until pH reached at 7 followed by placed in a vacuum.

Decoration of Ag nanoparticles as a sensing material was developed by two-step electroless deposition, which consists of one to form Ag seeds on the MWCNTs and another to grow the Ag nanoparticles. Firstly, the Ag seed was deposited using the Sn-induced selective electroless deposition process reported by Lee et al. (2005) and Chang et al. (2010). The MWCNTs deposited PTFE substrate was soaked in the mixed solution of 0.02 M SnCl_2_ (Daejung chemicals) and 0.02 M HCl (35%, Daejung chemicals) for 2 min at room temperature. The specimen was rinsed in acetone, ethanol, and deionized water and dried at room temperature. Reduction of Ag seed was performed in the electrolyte containing of 0.02 M AgNO_3_ (Sigma-aldrich) for 2 min. During this reaction, the Ag seeds were selectively reduced on sites of absorbed Sn. After rinsing, the growth of Ag nanoparticles was conducted in the electrolyte containing of 0.01 M AgNO_3_ and 0.1 M L-ascorbic acid as a reducing agent.

As an effective adsorption layer, copper benzene-1,3,5-tricarboxylate (Cu-BTC) was synthesized by a simple filtration method on the Ag-NPs decorated MWCNT/PTFE substrate (Stock and Biswas, 2012). The specimen was exposed to 0.15 M Cu(NO_3_)_2_3H_2_O (Sigma-aldrich) and 0.09 M C_9_H_6_O_6_ (Sigma-aldrich) ethanol solutions by terns. Rinsing with ethanol were performed between the steps. After the procedures, the as-synthesized specimen was placed into the vacuum chamber. The size of the sensor specimen was 1 × 1 cm. The morphology, crystallinity, and surface elements of specimens was investigated by field emission scanning electron microscope (FE-SEM; MIRA3, TESCAN), X-ray diffractometer (XRD; Rigaku SmartLab, Rigaku), and energy dispersive X-ray spectroscopy (EDS, EDAX TSL) analysis and x-ray photoelectron spectroscopy (XPS, ESCALAB250), respectively.

### Chloride Ion Sensing by Flexible Ag-NPs/AgCl Electrode Sensor

All sensing experiments were performed at room temperature and 6–7 pH except the one for sensing response in 13 pH. Concentration of chloride ions was varied from 0.001 to 0.3 M, and solution volume was fixed as 200 mL. Selectivity of the chloride ion sensor was tested by measuring its response toward various interfering ions such as potassium nitrate, sodium sulfate, sodium sulfide, and sodium hydroxide (Sigma-aldrich).

The electrical contact of the sensor was made by Ag paste shown in Figure S5. Chronoamperometry was used to monitor chloride ion sensing performance of the fabricated sensor. The applied voltage was selected to 1 V after careful observation of applied voltage effect on the sensing signal. For instance, when the applied voltage was >1 V, specimen was damaged because of joule heat evolved by high electrical potential and when the voltage was smaller than 1 V, the sensing signal was not detectable due to covered by noise level. Digital source meter (2636A, Keithley) was employed to supply the constant voltage and measure the change of signal current due to chloride-sensor element interactions. Experimental data were collected and displayed by labview software in monitoring computer *in situ*. The fabricated sensor device shows ohmic contact in Figure S6.

## Results and Discussion

### Fabrication of Flexible Ag-NPs/AgCl Electrode Sensor

**Figure 2:**
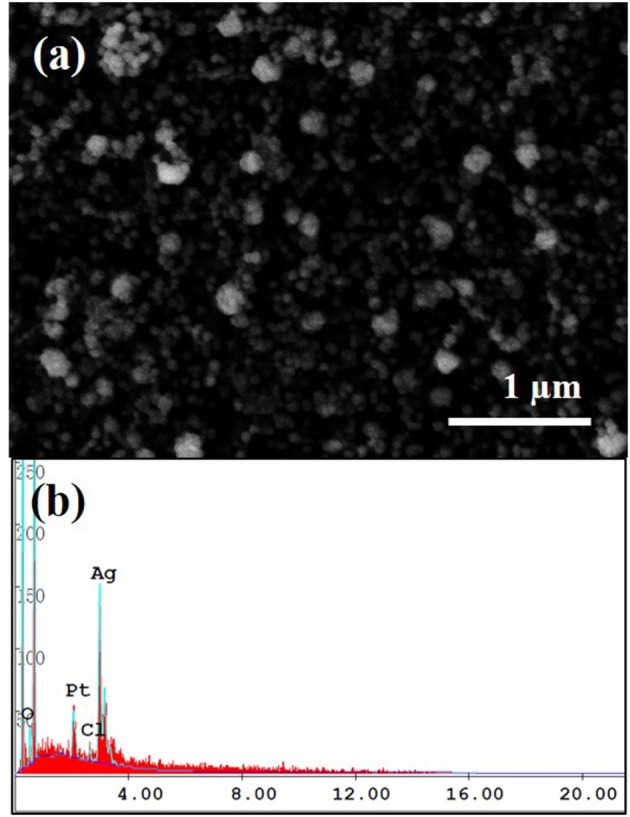
SEM image of Ag nanoparticles decorated on MWCNT **(a)** and its EDS spectrum **(b)**. The experiment was conducted in the electrolyte contained 0.01 M AgNO_3_ and 0.1 M L-ascorbic acid for 1 min at room temperature.

**Figure 3:**
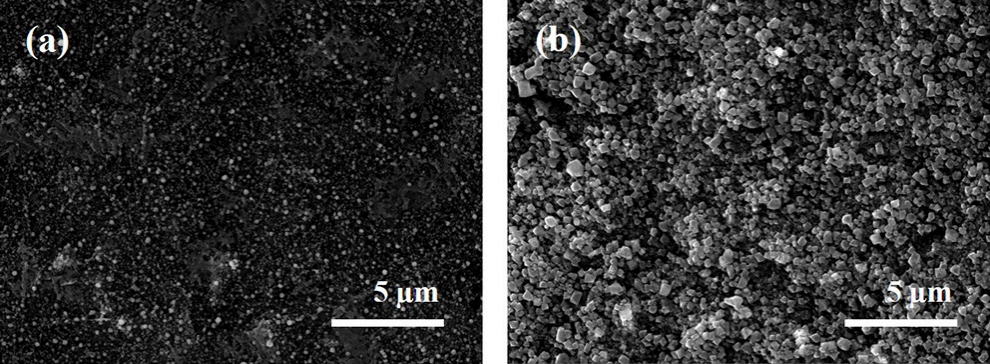
SEM image of Ag-NPs/MWCNT **(a)** and Cu-BTC coated Ag-NPs/MWCNT **(b)**.

The effect of electroless deposition time on the size and density of Ag nanoparticles was investigated by FE-SEM shown in Figure S1. The reaction was conducted in the electrolyte containing of 0.01 M AgNO_3_ and 0.1 M L-ascorbic acid as a reducing agent at room temperature. The deposited Ag nanoparticles were confirmed by EDS analysis shown in Figure 2. Morphology of Ag nanoparticles formed on the MWCNTs was typically globular with a dimeter of 50~120 nm (Figure S2). From this observation, the mean size of Ag nanoparticles increased by the reaction time, however, the big clusters were observed more often in the samples synthesized at longer reaction time. Accordingly, this agglomeration behavior became much severe as the reaction time increased, eventually, the Ag nanoparticles were detached out from the MWCNTs. The suitable reaction time chosen for the sensor fabrication was of 1 min in the electrolyte containing of 0.01 M AgNO_3_ and 0.1 M L-ascorbic acid which represented the securely-decorated Ag nanoparticles on the MWCNTs without deterioration (**Figure 3a**).

So as to develop the chloride ion sensor with the proper adsorption capacity and selectivity toward the chloride ions, the sensing electrode materials need to show good hydrophilicity. Therefore, to provide this property, metal-organic frameworks (MOFs), which consisted of self-assembly of organic ligands and metal oxide clusters, are applied where the partial positive charges on the metal sites in MOFs have the potential to enhance general adsorption properties (Karra and Walton, 2008). Moreover, the great flexibility and stability of MOFs is expected for its practical use in various application (Hamon et al., 2011). As the effective adsorption layer, copper benzene-1,3,5-tricarboxylate (Cu-BTC) was synthesized by a simple filtration method on the Ag-NPs decorated MWCNT/PTFE substrate. Surface morphology of the Cu-BTC coated Ag-NPs/MWCNT was investigated by SEM analysis. A clear octahedral structure of Cu-BTC with a size of ~500 nm was observed in Figure 3b which is consistent with the other result (Kang et al., 2018). High porosity of the Cu-BTC would help to hold the chloride ions in the electrolyte more effectively. The XRD patterns confirmed that the Cu-BTC was formed without destruction of Ag-NPs on the MWCNT (Figure S3; Houk et al., 2009; Saleh, 2013; Agasti and Kaushik, 2014; Khoshhal et al., 2015).

The XPS analysis indicated high resolution Cu2p, Ag3d, and C spectra data of Cu-BTC coated Ag-NPs/MWCNT (Figure S4). The Cu2p spectra were composed of four peaks centered at 935, 944, 955, and 963 eV, which correspond to the Cu2p1/2 peak, the satellite Cu2p1/2 peak, the Cu2p3/2 peak and satellite Cu2p3/2 peak, respectively. The Ag3d spectra were composed of two peaks at 368 and 374 eV, which correspond to the Ag3d5/2 and Ag3d3/2, respectively. The carbon peaks such as C–C (MWCNT, PTFE, Cu-BTC), C–O (carboxylic group on CNT surface and Cu-BTC), O=C–O (carboxylic group on CNT surface and Cu-BTC), and C-F_2_ (PTFE) were also confirmed.

**Figure 4:**
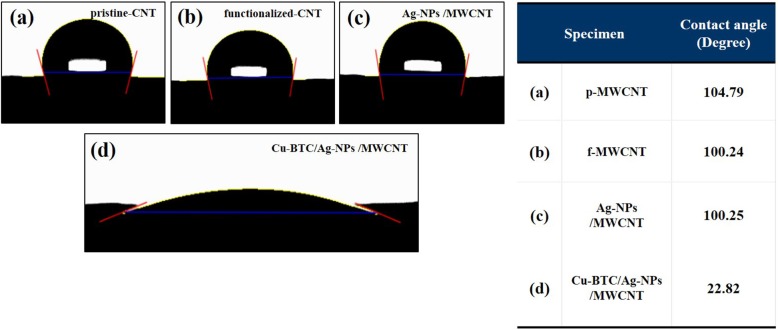
Contact angle measurement of pristine-CNT **(a)**, functionalized-CNT **(b)**, Ag-NPs /MWCNT **(c)**, and Cu-BTC/Ag-NPs /MWCNT **(d)**.

The effect of Cu-BTC on the wettability of the specimens was confirmed by contact angle measurement. The change of the degree of hydrophilicity was compared by the contact angle of water droplets. The slight decrease of contact angle of the functionalized-CNT (100.24°) was attributed to the existence of carboxyl and hydroxyl groups. The Cu-BTC coated specimen displayed distinctive hydrophilic property with wider contact angle (22.82°) whereby the pristine- and functionalized-CNT, and Ag-NPs decorated CNT shows relatively hydrophobic properties with the semicircle-like water droplet on the surfaces (Figure 4). Therefore, the increased wettability of the Ag-NPs/MWCNT sensor by the Cu-BTC coating process may enhance the adsorption of chloride ion on the Ag-NPs resulting the improved sensor performance.

### Sensor Performance of Flexible Ag-NPs/AgCl Electrode Sensor

**Figure 5:**
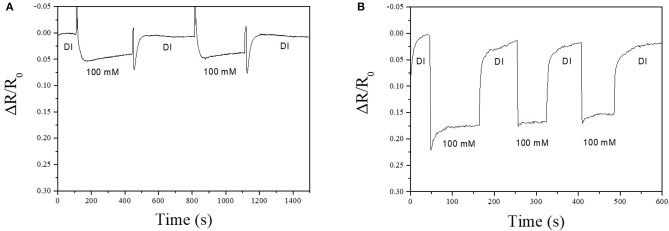
Chloride ion sensing response of Ag-NPs/MWCNT/PTFE **(A)** and Cu-BTC coated Ag-NPs/MWCNT/PTFE **(B)**. Electrolyte contains 100 mM potassium chloride at room temperature. pH was maintained at 6~7. Applied voltage was 1 V.

Sensing performance of the bare-MWCNT, Ag-NPs/MWCNT, and Cu-BTC coated Ag-NPs/MWCNT sensors toward the chloride ion was investigated. The fabricated sensor showed ohmic characteristic and base resistance was about 300 KΩ confirmed by the I-V measurement. The sensing response was observed with concentrations of chloride ion ranging from 1 to 300 mM at pH 6–7 at room temperature. The response was defined by the resistance change (ΔR/R_0_) where ΔR is the resistance change when the sensor was immersed in the test solution and A_0_ is the initial baseline resistance in the DI water. The response time was defined as the time to reach 90% of its steady-state value. The recovery time was defined as the time required for the sensor to return to 90% of its maximum response (Hideaki et al., 1995). The bare-MWCNT shows no response toward various concentration of chloride ions confirming that the sensing responses actually came from Ag-NPs (Figure S7). There was a clear sensing response of the Ag-NPs/MWCNT sensor at 100 mM potassium chloride with a response and recovery time of 45 and 58 s, respectively (Figure 5A). This sensor operation is explained by electron transport from chloride ion to MWCNTs. General mechanism is explained that when the sensor was exposed to chloride ion solution, the surface of Ag-NPs reacted with chloride ion is transformed to AgCl along with the generation of electrons (Montemor et al., 2006). This electron donation decreases the resistance of the sensor so that it reads as sensing signals. Interestingly, the Cu-BTC coated Ag-NPs/MWCNT device showed the increased sensing performance compared to that of the sensor without Cu-BTC coating (Figure 5B). The resistance clearly decreased much more than about 4 times with the fast response (4.5 s) and recovery (9.3 s) time. The enhancement of the sensing performance might be elucidated that the Cu-BTC layer would work as an effective medium between the Ag-NPs surface and electrolyte. The exposed surface of the sensor treated no Cu-BTC consisted of Ag-NPs but largely highly hydrophobic MWCNTs. Therefore, not only increased hydrophilicity the porous structure of Cu-BTC would hold the electrolyte for effective adsorption of analytes with large specific surface area (Li et al., 2018).

**Figure 6:**
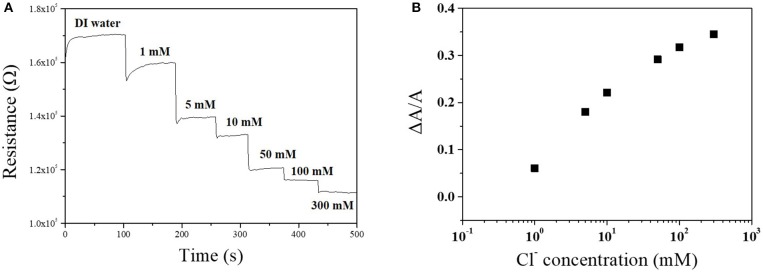
Chloride ion sensing response of Cu-BTC coated Ag-NPs/MWCNT/PTFE as a function of chloride ion concentration **(A)** and a change of current **(B)**. Electrolyte contains 1~300 mM potassium chloride at room temperature. pH was maintained at 6~7. Applied voltage was 1 V.

The sensitivity of the Cu-BTC coated Ag-NPs/MWCNT sensor toward chloride ion was characterized by the sensing test at various potassium chloride concentration. As the chloride ion concentration increased from 1 to 300 mM, the intensity of resistance change increased which demonstrated that the fabricated sensor is feasible in various conditions (Figure 6A). The sensitivity (ΔA/A) almost linearly increased up to 50 mM and slightly slow down at higher potassium concentrations (100 and 200 mM) (Figure 6B).

**Figure 7:**
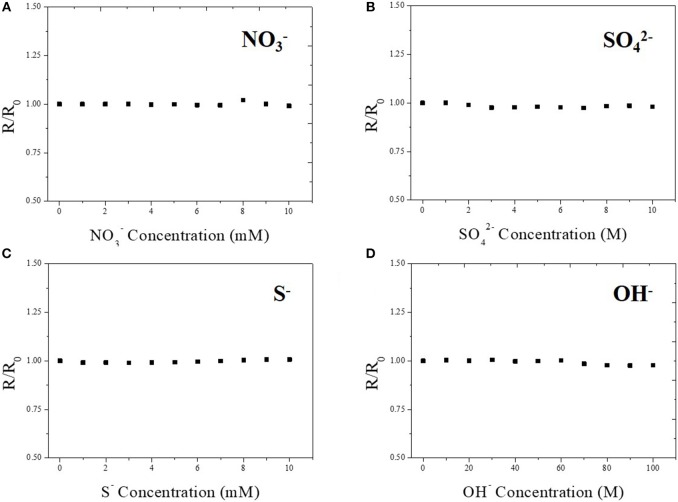
Selectivity test of Cu-BTC coated Ag-NPs/MWCNT/PTFE toward a variety of ions such as NO^3−^ **(A)**, ${\text{SO}}_{4}^{2-}$ **(B)**, S^−^ **(C)**, and OH^−^ **(D)**. Electrolyte contains 100 mM potassium chloride at room temperature. pH was maintained at 6~7. Applied voltage was 1 V.

The selectivity of the Cu-BTC coated Ag-NPs/MWCNT sensor is also tested to identify possible interferences for practical use. The sensitivity was determined by analyzing the response toward various ions such as NO^3−^, ${\text{SO}}_{4}^{2-}$, S^−^, and OH^−^. As shown in Figure 7, there was no signal detected from any possible interfering analytes indicating that the fabricated sensor has high selectivity toward only chloride ion. Additionally, we confirmed that the fabricated sensor worked at pH 13 in order to confirm its functionally in the general environment for commercial chloride ion sensors operation (Figure S8). The sensor works properly showing the increase in the current as a function of the chloride ion concentration, however, there is a detachment issue after operation. This limitation will be further studied as a future work to optimize the sensor in alkali environment.

## Conclusion

In this study, flexible chloride ion sensor based on MWCNT, silver nanoparticles, and Cu-BTC was investigated. All materials were deposited on flexible PTFE substrate. The MWCNT and Cu-BTC were deposited by vacuum aspirating, and silver nanoparticles were synthesized by two-step electroless deposition. The fabricated sensor was studied by using scanning electron microscopy, energy dispersive spectrometry, x-ray photoelectron spectroscopy, x-ray diffraction, and all intended materials were investigated. Sensing experiments were analyzed by chronoamperometric method. As a result, possibility of chloride ion detection of Ag-NPs/MWCNT sensor was identified, but the sensor performance was not enough for practical use. To solve this problem, Cu-BTC layer was coated on the surface of Ag-NPs/MWCNT sensor because of its high hydrophilicity and large surface area, and the enhancement of sensor performance was verified by results of decreased response/recovery time and sensitivity. Also the selectivity of Cu-BTC coated Ag-NPs/MWCNT sensor was identified by sensing tests with electrolyte including interfering ions.

## Data Availability Statement

All datasets generated for this study are included in the manuscript/Supplementary Files.

## Author Contributions

BY, H-SL, and JK contributed conception and design of the study. BK organized the database. BK, JK, and SP performed the statistical analysis. JK and BK wrote the first draft of the manuscript. All authors contributed to manuscript revision, read, and approved the submitted version.

### Conflict of Interest

The authors declare that the research was conducted in the absence of any commercial or financial relationships that could be construed as a potential conflict of interest. The handling editor declared a shared affiliation, though no other collaboration, with the authors BY, BK, SP, and H-SL at the time of the review.

## Supplementary Material

Figure S1 Size dependence of Ag nanoparticles as a function of electroless deposition time. The reaction was conducted at room temperature in neutral environment. 10 **(a)**, 60 **(b)**, 300 **(c)**, and 600 s **(d)**.

Figure S2 Diameter of Ag nanoparticles as a function of electroless deposition time. The reaction was conducted at room temperature in neutral environment.

Figure S3 XRD analysis of MWCNT/PTFE **(a)**, Ag-NPs/MWCNT/PTFE **(b)**, and Cu-BTC coated Ag-NPs/MWCNT/PTFE **(c)**.

Figure S4 XPS analysis of Cu-BTC/Ag-NPs/MWCNT. C peak **(a)**, Ag peak **(b)**, and Cu peak **(c)**.

Figure S5 Schematic cross sectional view of a flexible Ag/AgCl electrode sensor.

Figure S6 I-V curve of a flexible Ag/AgCl electrode sensor.

Figure S7 No sensing response of the bare MWCNT/PTFE substrate toward 100 and 500 mM of potassium chloride at room temperature. Applied voltage was 1 V.

Figure S8 Chloride ion sensing response of Cu-BTC coated Ag-NPs/MWCNT/PTFE as a function of chloride ion concentration at pH 13 **(a)** and a change of current depending on the chloride ion concentration **(b)**. Electrolyte contains 1, 10, and 100 mM potassium chloride, 0.1 M hydroxide at room temperature. Applied voltage was 1 V.
