# Supplementary figures and images for: Improved Chloride Ion Sensing Performance of Flexible Ag-NPs/AgCl Electrode Sensor Using Cu-BTC as an Effective Adsorption Layer
Source: Front Chem. 2019 Sep 24;7:637. doi: 10.3389/fchem.2019.00637 (PMC6768955; doi:10.3389/fchem.2019.00637)

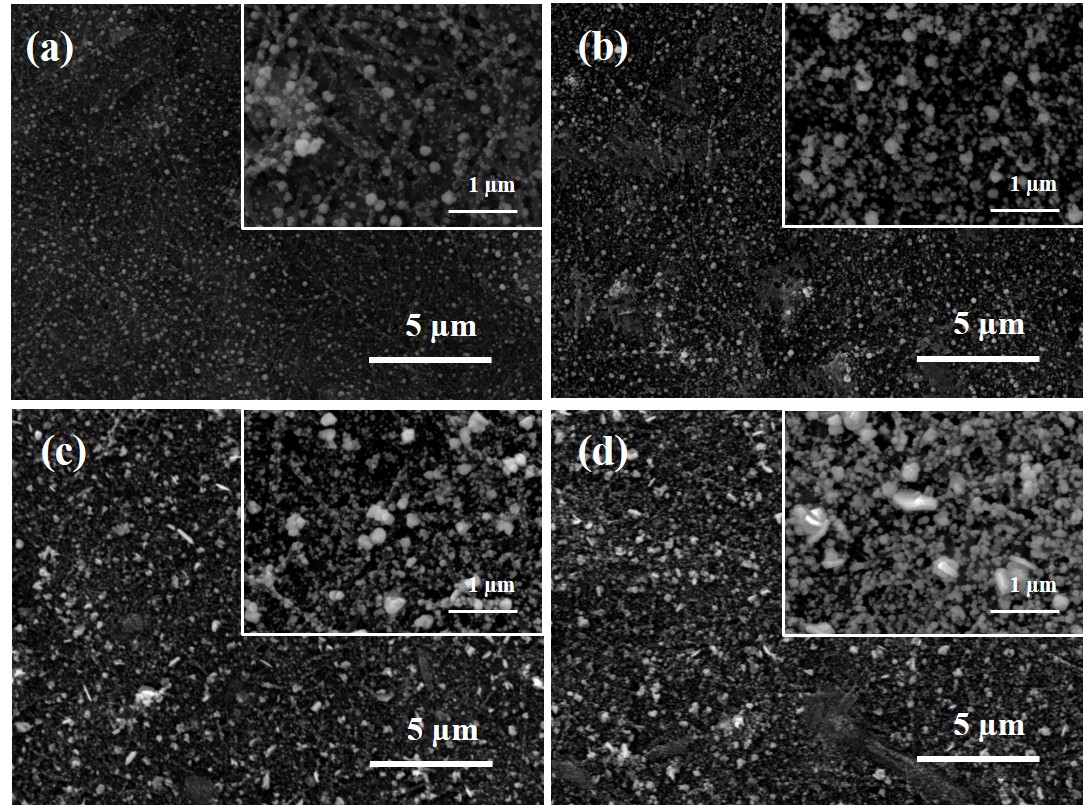

Supplement: Figure S1 — Size dependence of Ag nanoparticles as a function of electroless deposition time. The reaction was conducted at room temperature in neutral environment. 10 (a), 60 (b), 300 (c), and 600 s (d). [file Image_1.jpeg]

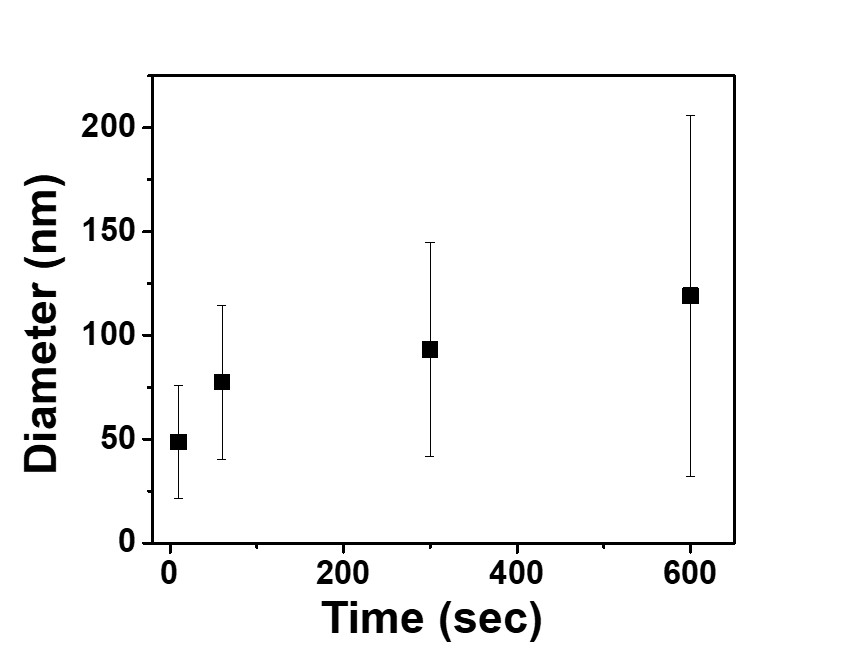

Supplement: Figure S2 — Diameter of Ag nanoparticles as a function of electroless deposition time. The reaction was conducted at room temperature in neutral environment. [file Image_2.jpeg]

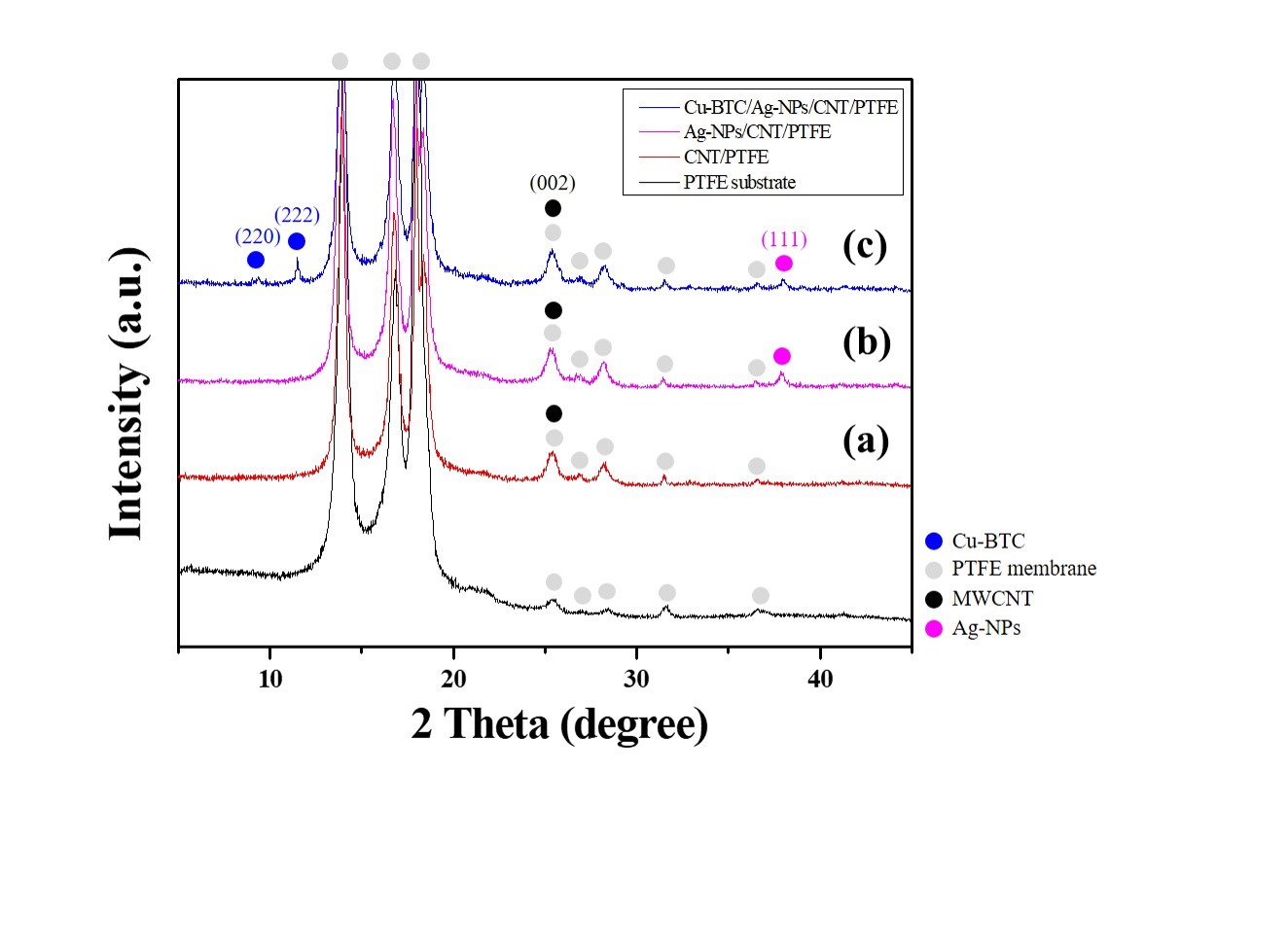

Supplement: Figure S3 — XRD analysis of MWCNT/PTFE (a), Ag-NPs/MWCNT/PTFE (b), and Cu-BTC coated Ag-NPs/MWCNT/PTFE (c). [file Image_3.jpg]

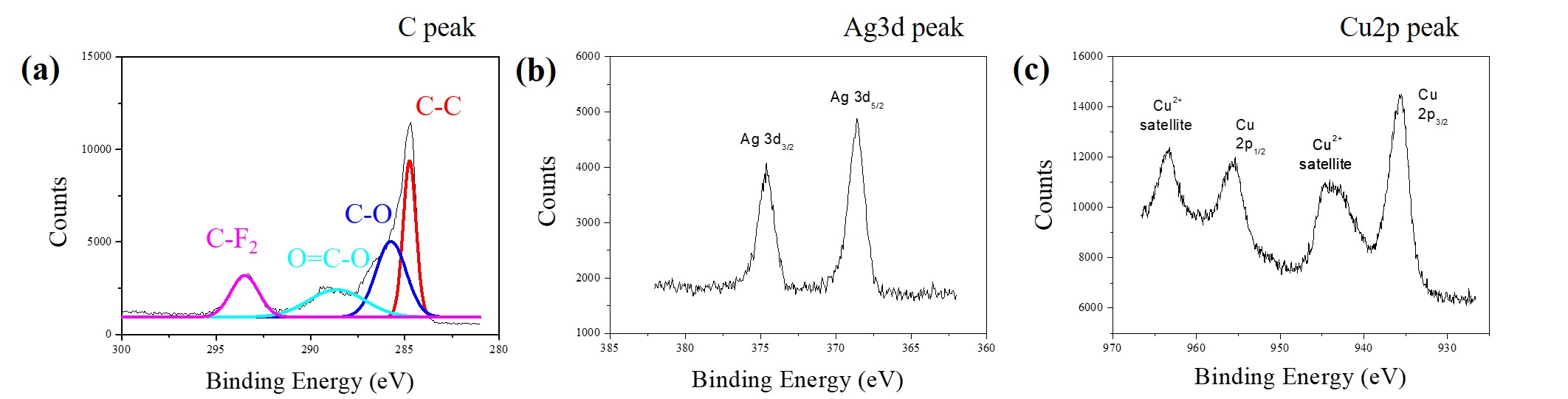

Supplement: Figure S4 — XPS analysis of Cu-BTC/Ag-NPs/MWCNT. C peak (a), Ag peak (b), and Cu peak (c). [file Image_4.jpeg]

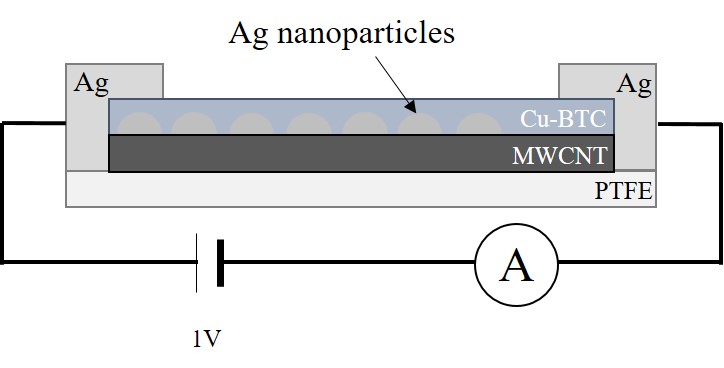

Supplement: Figure S5 — Schematic cross sectional view of a flexible Ag/AgCl electrode sensor. [file Image_5.jpeg]

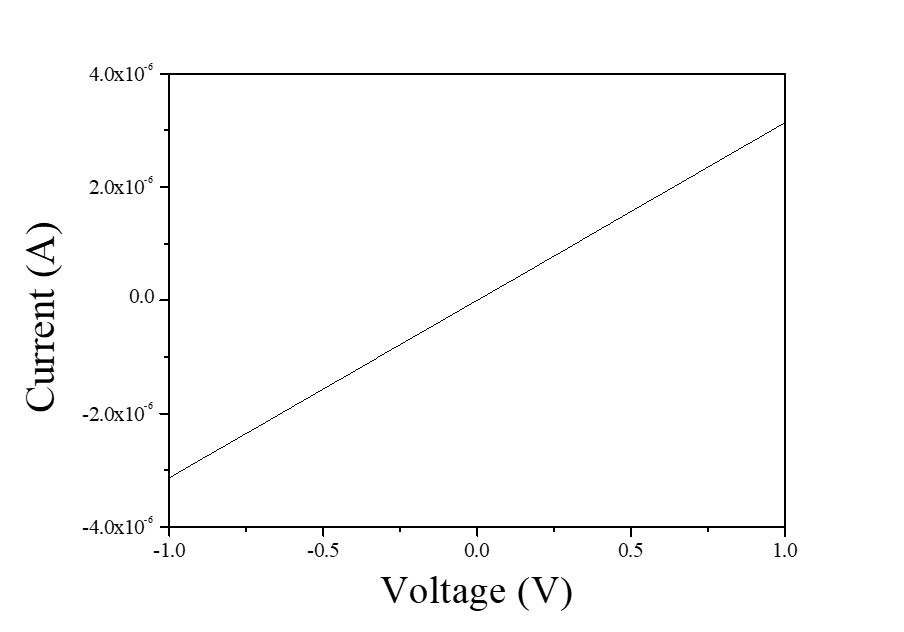

Supplement: Figure S6 — I-V curve of a flexible Ag/AgCl electrode sensor. [file Image_6.jpeg]

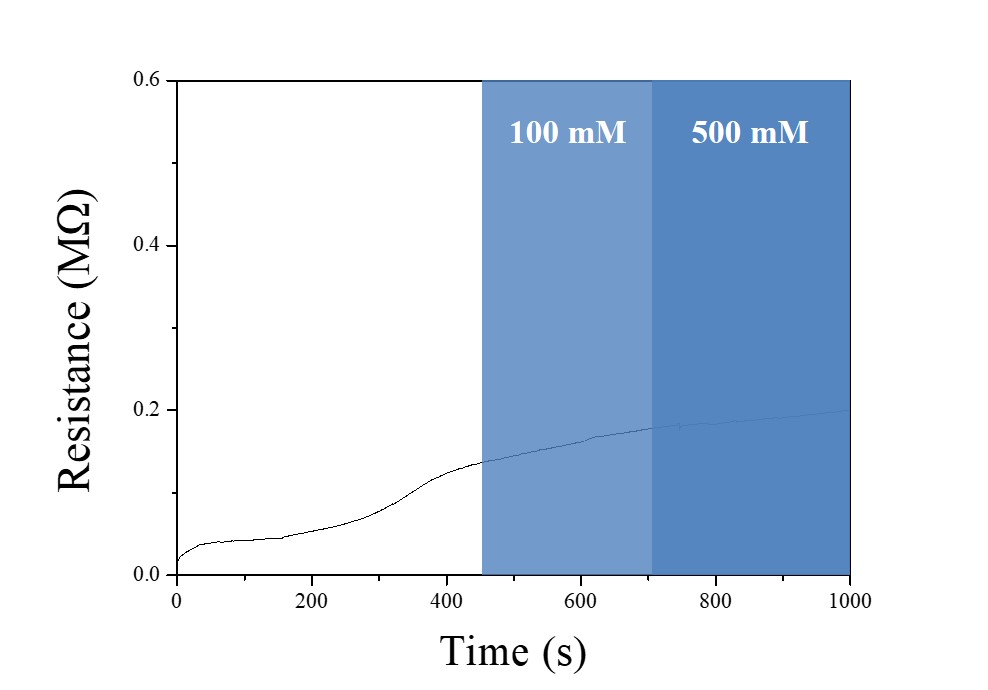

Supplement: Figure S7 — No sensing response of the bare MWCNT/PTFE substrate toward 100 and 500 mM of potassium chloride at room temperature. Applied voltage was 1 V. [file Image_7.jpeg]

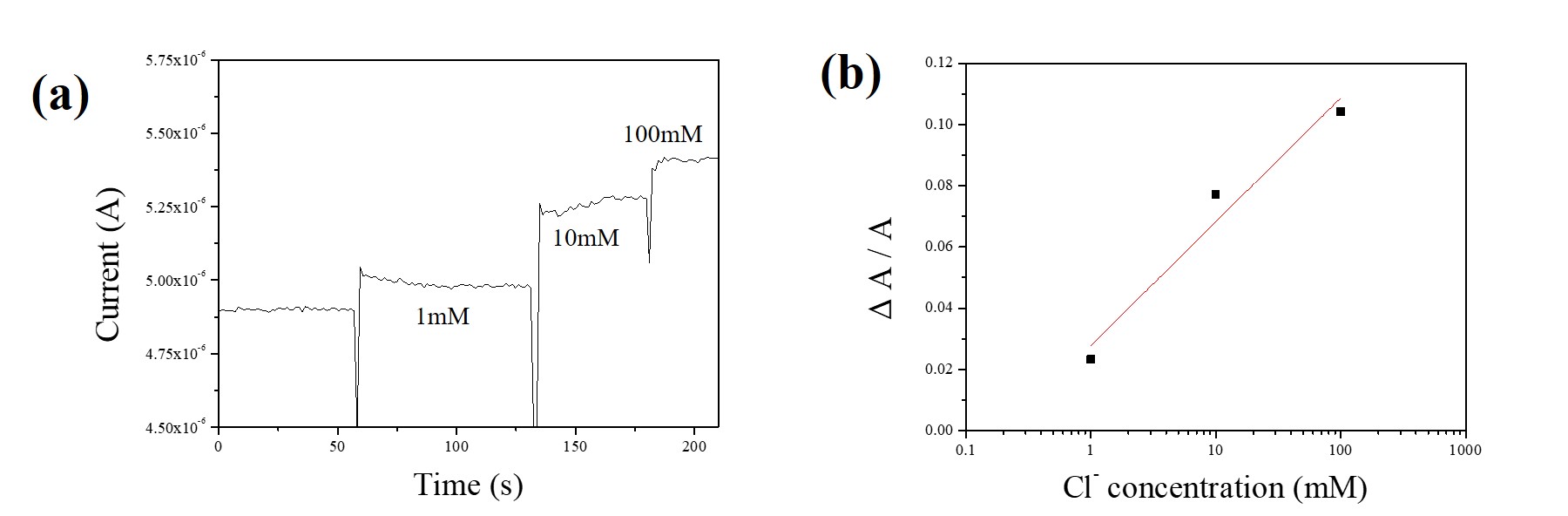

Supplement: Figure S8 — Chloride ion sensing response of Cu-BTC coated Ag-NPs/MWCNT/PTFE as a function of chloride ion concentration at pH 13 (a) and a change of current depending on the chloride ion concentration (b). Electrolyte contains 1, 10, and 100 mM potassium chloride, 0.1 M hydroxide at room temperature. Applied voltage was 1 V. [file Image_8.jpeg]
